# Magnetic resonance flow velocity and temperature mapping of a shape memory polymer foam device

**DOI:** 10.1186/1475-925X-8-42

**Published:** 2009-12-31

**Authors:** Ward Small, Erica Gjersing, Julie L Herberg, Thomas S Wilson, Duncan J Maitland

**Affiliations:** 1 Lawrence Livermore National Laboratory, Livermore, California, 94550, USA; 2 Department of Chemical Engineering and Materials Science, University of California, Davis, California, 95616, USA; 3 Department of Biomedical Engineering, Texas A&M University, College Station, Texas, 77843, USA

## Abstract

**Background:**

Interventional medical devices based on thermally responsive shape memory polymer (SMP) are under development to treat stroke victims. The goals of these catheter-delivered devices include re-establishing blood flow in occluded arteries and preventing aneurysm rupture. Because these devices alter the hemodynamics and dissipate thermal energy during the therapeutic procedure, a first step in the device development process is to investigate fluid velocity and temperature changes following device deployment.

**Methods:**

A laser-heated SMP foam device was deployed in a simplified *in vitro *vascular model. Magnetic resonance imaging (MRI) techniques were used to assess the fluid dynamics and thermal changes associated with device deployment.

**Results:**

Spatial maps of the steady-state fluid velocity and temperature change inside and outside the laser-heated SMP foam device were acquired.

**Conclusions:**

Though non-physiological conditions were used in this initial study, the utility of MRI in the development of a thermally-activated SMP foam device has been demonstrated.

## Background

Shape memory polymers (SMPs) are a class of polymeric materials that can be fabricated into a primary shape, deformed into a stable secondary shape, and controllably actuated to recover the primary shape. The basis for the shape memory effect has been previously described in detail [1]. Although there is wide chemical variation in these materials, they can be grouped into categories with high physical similarity based on the method of actuation, which can be achieved thermally, through photo-induced reaction, or by introduction of an external plasticizer [2]. For SMPs that are actuated thermally, such as those in the present work, raising the temperature of the polymer above its characteristic glass transition temperature (T_g_) results in a decrease in the elastic modulus from that of the glassy state (~10^9 ^Pa) to that of an elastomer (~10^6 ^to 10^7 ^Pa) [3] as the primary shape is recovered. Upon cooling, the original modulus is nearly completely recovered and the primary form is stabilized [4].

Encouraged by the shape memory behavior and biocompatibility [5,6], many biomedical applications for SMP-based active devices have emerged [2]. In particular, researchers are developing various interventional medical devices based on thermally responsive SMP. Such catheter-delivered devices include expandable stents [7,8], microactuators for retrieving blood clots in ischemic stroke patients [9,10], and embolic coils [11] and foams [12,13] for filling aneurysms.

When the T_g _of the SMP is above body temperature (37°C), an external heating mechanism such as laser (photothermal) [9,14] or electroresistive [10,14] heating is needed. Safe and effective device actuation requires limiting the thermal impact to the surrounding blood and tissue, posing a key challenge in SMP interventional device development. Another development consideration relevant for implantable devices (e.g., stent or embolic device) is the effect of the deployed device on the blood flow. Since changes in the hemodynamics and temperature induced by the intervention ultimately govern its safety and efficacy, there is a need to understand these changes and their physiological impact. A first step in the device development process is to investigate fluid velocity and temperature changes following device deployment in a simplified *in vitro *model. Though the results of such an investigation do not necessarily provide a direct assessment of the physiological impact in an actual clinical procedure, they may be used to modify device properties (e.g., foam density), adjust heating parameters (e.g., laser power), and validate computational models which can be extended to simulate physiological conditions.

The non-invasive methods of nuclear magnetic resonance (NMR) and magnetic resonance imaging (MRI) have been extensively used for chemical, material, biological, and medical applications. In MRI methods, a magnetic field gradient is used in conjunction with an NMR experiment to spatially encode spectral signatures based on numerous contrast parameters. These signatures may include structure (chemical shift), dynamics (relaxation times), or velocity (diffusion and flow). Spatial maps of fluid flow and temperature obtained by MRI can provide insight into the impact of the intervention.

Our team has previously reported the use of various tools to study the fluid and thermal dynamics associated with the deployment of SMP devices under development. These tools include computational fluid dynamics models [15], flow visualization and particle image velocimetry [16], and contact temperature measurement [13]. The primary aim of this study is to demonstrate the utility of non-invasive MRI techniques to aid the early stage development of an SMP embolic foam device. We describe the use of established MRI methods to quantify the steady-state fluid flow and temperature during laser heating of a generic, non-clinical SMP embolic foam device in constant water flow in a straight tube. The SMP foam device, laser heating scheme, flow system, and MRI system are described, and the flow velocity and temperature change inside and outside the foam device are reported. The benefits of non-invasive MRI for preliminary device development are discussed.

## Methods

### SMP Foam Device

The chemically blown foam was based on SMP developed at Lawrence Livermore National Laboratory (LLNL) comprised of hexamethylene diisocyanate, N, N, N', N'-tetrakis(2-hydroxypropyl)ethylenediamine, and triethanolamine [3]. Dye (Epolight™ 4121, Epolin, Inc.) was added during processing to aid laser light absorption. The predominantly open cell foam had a T_g _of ~45°C, dye concentration of ~900 ppm (absorption coefficient ~10 cm^-1^), density of 0.020 g/cc, cell size of ~200-300 μm, and calculated volumetric void fraction of 98.4%, allowing for a theoretical volume expansibility from a fully collapsed state of 60×. Different foam properties, including T_g_, may be achieved by adjusting the chemical composition.

A key property of the SMPs is their ability to maintain a secondary shape that is different than the original primary shape. The secondary shape is obtained by reforming the SMP while heated above T_g _and then cooling it to stabilize the new shape. For the SMP foams, the secondary shape is obtained by compressing the foam to reduce its volume (a key feature enabling catheter delivery of the device). Heating near T_g _will induce shape recovery (expansion). In the case of SMP foams, the transition from the secondary to the primary shape is not sharp like a crystalline melting point; the elastic modulus falls gradually over a span of ~30°C with the nominal T_g _approximately centered in the decline.

**Figure 1:**
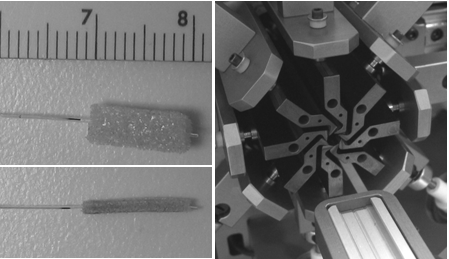
**SMP foam device**. SMP foam device on the light diffusing fiber in its expanded form (top left). The fiber is threaded axially through the foam cylinder. Eight-blade cylindrical crimping machine with heated blades (right) used to collapse the foam into its secondary form (bottom left). Laser light emitted from the light diffusing fiber heats the surrounding collapsed foam, causing it to expand (photothermal actuation). Minor scale divisions in millimeters.

The SMP foam device used in this study was made specifically for use in the straight tube geometry, and does not necessarily represent a clinically-relevant design. Unlike the spherically shaped devices used previously to demonstrate the concept of aneurysm occlusion *in vitro *[13], the foam was cut into a 4-mm-diameter by 10-mm-long cylinder (using a biopsy punch). The foam was threaded coaxially over a 300-μm-diameter by 10-mm-long cylindrical light diffusing fiber (made in-house from LLNL SMP [17]) and collapsed using a crimping machine (Model W8FH, Interface Associates) at 93°C. Upon reaching the collapsed diameter (1 mm) the device was allowed to cool to room temperature and then released from the machine. The device is shown in the expanded and collapsed forms in Fig. 1.

To fabricate the diffusing fiber, LLNL SMP (T_g_~81°C) was cast in a teflon tube (inner diameter = 300 μm) over a 100-μm-core cleaved optical fiber. The resulting SMP rod was then media blasted with 100-μm sodium bicarbonate particles to create a diffusing surface. A ST connector was added to the proximal end of the optical fiber for coupling to the laser light source, an 810-nm continuous-wave diode laser pigtailed into a 100-μm-core silica optical fiber (Model UM7800/100/20, Unique Mode). Nearly 90% of the light was emitted radially, with the remaining light emerging from the distal end of the diffuser. The SMP formulation was specifically designed to be optically transparent at the laser wavelength (absorption coefficient = 0.01 cm^-1 ^at 810 nm).

### Flow System

**Figure 2:**
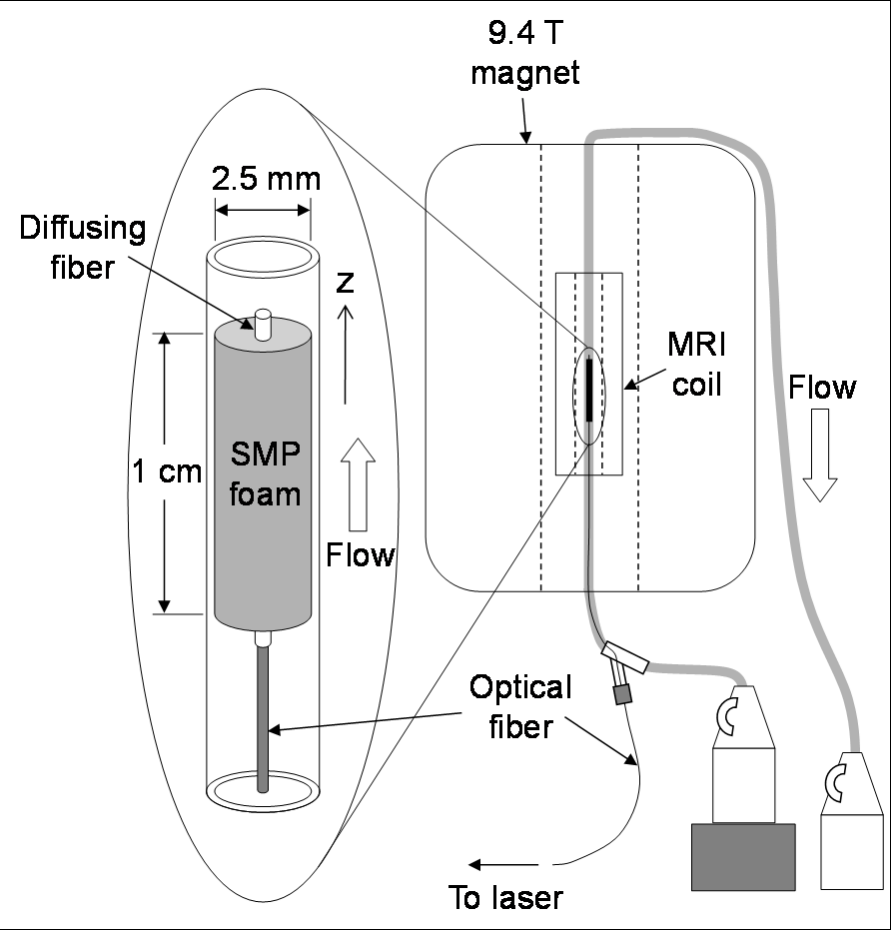
**Flow system**. Closed flow system. Water at room temperature flowed at 3.8 ml/min (~13 mm/s) through the silicone tubing from the elevated container to the surface container. The silicone tubing was fed through the MRI coil which was located inside the 9.4 T magnet. The SMP foam device was inserted into the tubing via a Touhy Borst valve and positioned inside the MRI coil. Water flow inside the MRI coil is in the z-direction.

The flow system is shown in Fig. 2. Silicone tubing (inner diameter = 2.5 mm) was fed through the MRI coil which was positioned inside the magnet. The two ends of the tubing were placed in two separate containers holding water at room temperature, forming a closed system. One of the containers was elevated, causing water to flow through the tubing at a constant rate of 3.8 ml/min (~13 mm/s). The SMP foam device mounted on the diffusing fiber was inserted into the tubing via a Touhy Borst valve and positioned inside the MRI coil. The optical fiber served as the transport vehicle to deliver the device into the tubing; a guiding catheter was not used. The device position was adjusted to acquire images inside and outside the SMP foam.

### MRI Experimental Parameters

Images were acquired at 9.4 T with a Bruker Avance spectrometer and a Bruker Micro2.5 microimaging probe equipped with a 5-mm coil. The fluid used in all experiments was ionized water doped with a small amount (0.3 ml/L) of MRI contrast agent (Magnevist, Berlex Laboratories) so that faster repetition time (TR) could be used, which shortens the experimental scan time. Images of the area occupied by the foam were acquired using a spin echo imaging sequence with a field of view (FOV) of 2 × 2 cm, imaging matrix of 128 × 128 (156 × 156 μm pixel resolution), TR of 400 ms, echo time (TE) of 7 ms, and an experiment acquisition time of 51 s. Fluid flow was turned off to acquire these images.

Fluid flow images, depicting the three-dimensional velocity patterns, were acquired using a diffusion weighted, spin echo, phase encoding sequence. Four slices were acquired for each scan with a thickness of 2 mm and FOV = 0.5 × 0.5 cm, imaging matrix = 128 × 128 (39 × 39 μm pixel resolution), TR = 400 ms, and TE = 15.2 ms. For flow encoding, the following parameter values were used: Δ*t *= 7 ms, *δ *= 1 ms, and *g *= 0 and 5 mT/m, where Δ*t *is the time between the two motion encoding gradients, *δ *is the duration of the motion encoding gradients, and *g *is the strength of the gradients. The resulting scan time was 2 min and 8 s. After subtracting the phase images from the two different *g *values, the phase difference, Δ*φ*, was converted to velocity, *V*, using the formula *V *= Δ*φ*/(*δ*Δ*tγg*), where *γ *is the gyromagnetic ratio for protons (42.58 MHz/T) [18]. In order to fully characterize the flow, three velocity encoded images were obtained, one for each spatial dimension, using the same parameters described above. Images were acquired using a fluid flow rate of 3.8 ml/min.

There exist several methods of measuring the temperature with MRI, including observing changes in spin-lattice relaxation (T_1_), determining the diffusion constant over a specific region, or through the proton resonance frequency (PRF). The PRF method for determining temperature with MRI is the method of choice because it is independent of tissue composition, unlike measuring temperature with MRI techniques, such as spin-lattice relaxation time and the diffusion times, which are dependent on tissue composition. PRF utilizes the average hydrogen bond strength and can be determined from the phase in gradient echo strength, which is based on the chemical shift properties. The chemical shift is the separation of resonance frequencies from an arbitrary chosen resonance frequency and is caused by the shielding effects of the electronic environment of the nucleus. This means that nuclei in different chemical environments give rise to MRI signals at different frequencies. Since the chemical shift depends on temperature, changes in the strength of hydrogen bonding causes changes in chemical shifts, which can be used to measure changes in temperature. For these reasons, we used a gradient echo sequence. The gradient echo sequence was optimized by maximizing TE, TR, and gradient properties.

A gradient echo, phase encoding pulse sequence was employed to obtain spatial temperature maps. Acquisition parameters of TR = 90 ms, TE = 10 ms, and flip angle = 45° were used. Two slices were acquired during each 46-s scan with a FOV = 1 × 1 cm, imaging matrix = 128 × 128 (78 × 78 μm pixel resolution), and a slice thickness of 2 mm. Temperature maps were obtained from the phase difference between an image at a reference temperature and an image with laser heating, with fluid flow turned on for both. The relationship between phase difference, Δ*φ*, and temperature change, Δ*T*, is given by Δ*T *= Δ*φ*/(*αγB*_0_*T*_E_), where *α *is the thermal coefficient (0.01 ppm/°C in water), *B*_0 _is the magnetic field strength, and *T*_E _is the echo time of the experiment [19-21]. Temperature images were acquired with laser powers of 0, 1, 3, 5 and 7 W and a fluid flow rate of 3.8 ml/min.

Steady-state x, y, and z components of the fluid velocity (with z along the tubing in the direction of the main flow) were acquired immediately proximal, inside, and distal to the expanded SMP foam. Steady-state fluid temperature images were acquired immediately proximal to and inside the expanded SMP foam. Only measurements of the fluid were made; the MRI technique is not sensitive to temperature changes of the silicone tubing, SMP, or silica optical fiber. Due to the relatively long scan time (~2 min), no images were acquired during the initial laser-induced expansion of the SMP foam. While it may be possible to slightly increase the temporal resolution, the MRI system is not capable of imaging the continuously changing flow and temperature profiles as the foam expands.

## Results

**Figure 3:**
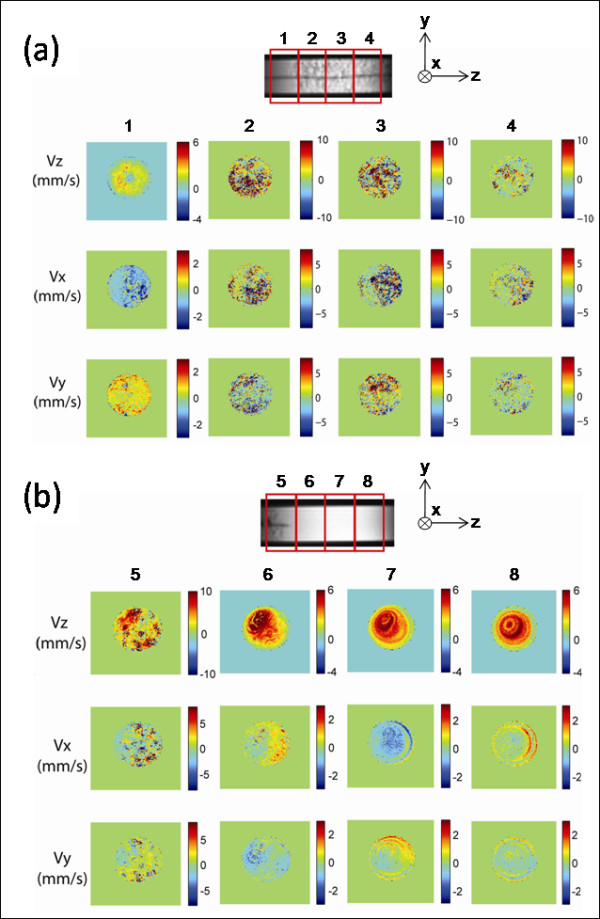
**Flow velocity**. Components of the steady-state fluid flow velocity in the (a) proximal and (b) distal regions of the expanded SMP foam device acquired by MRI. The main flow is from left to right in the z-direction. Each column corresponds to a 2-mm slice as denoted by the numbered boxes overlaying the image of the area occupied by the foam device. The flow becomes faster and less forward-directed inside the foam, then slows and becomes more forward-directed upon exiting the foam.

Steady-state fluid velocity images immediately proximal to and inside the expanded SMP foam are shown in Fig. 3(a), and distal to the foam in Fig. 3(b). Also shown are images of the area occupied by the foam device; the speckled region corresponds to the foam and the dark horizontal line corresponds to the coaxial diffusing fiber. The flow velocity initially increases and becomes less forward-directed inside the foam as the water is forced through the open cell structure. The circular zero-flow area approximately centered in the foam corresponds to the 300-μm-diameter diffusing fiber. Because the foam is still partially compressed in the tubing (original foam diameter = 4 mm, tubing inner diameter = 2.5 mm), the cell size is generally less than that of the fully expanded foam (~200-300 μm). The foam structure is evident in the speckled pattern of the flow velocity images inside the foam. Because the images represent average values over an axial distance of 2 mm, the speckles do not correspond to actual cells in the foam. The velocity decreases after exiting the foam and becomes more forward-directed.

**Figure 4:**
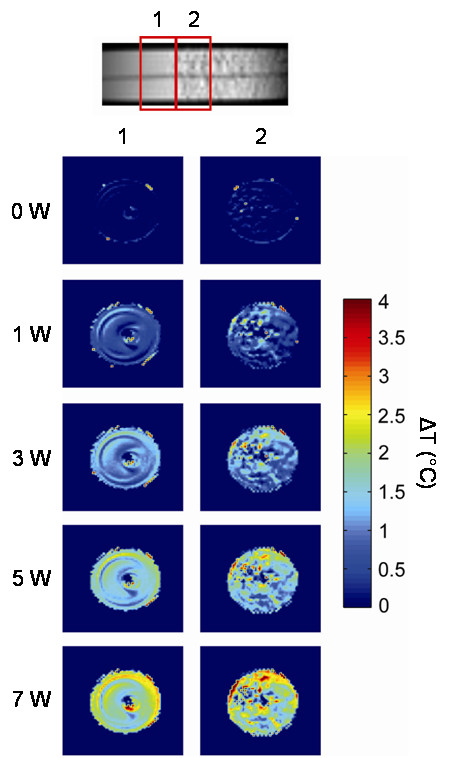
**Temperature change**. Steady-state fluid temperature change before (proximal to) and inside the expanded SMP foam during laser heating acquired by MRI. Images at laser powers of 0, 1, 3, 5, and 7 W are shown. Each column corresponds to a 2-mm slice as denoted by the numbered boxes overlaying the image of the area occupied by the foam device. Higher laser powers resulted in higher temperatures inside and outside the foam, with a maximum temperature change of 4°C at 7 W.

Steady-state fluid temperature images at laser powers from 0 to 7 W immediately proximal to and inside the expanded SMP foam are shown in Fig. 4. The temperature of the fluid inside the proximal end of the foam increases with laser power due to heat transfer from the laser-heated foam, reaching a maximum temperature change of 4°C at 7 W. Heat transfer to the fluid outside the foam is also evident. Flow artifacts are present in the images, especially in the phase-encode direction. The appearance of concentric rings of higher temperature at the boundary of the SMP and containing tube are most likely due to these flow artifacts. In a gradient echo sequence, the flow compensation techniques, such as gradient moment nulling, can be used to eliminate this problem. This will be implemented in future studies.

## Discussion

Given the sub-physiological fluid flow rate (blood flow in the basilar artery is ~70-170 ml/min during the cardiac cycle [22]), non-pulsatile flow, sub-37°C water temperature, use of water instead of blood, and lack of a mock aneurysm in the flow system, the *in vitro *model was not intended to accurately represent actual physiological conditions. Consequently, the measured values are not necessarily expected to represent those encountered in a clinical scenario. However, under certain conditions the data may be somewhat relevant. For example, an actual clinical device could include a baffle or other means of temporarily reducing the flow (e.g., balloon catheter) to facilitate thermal actuation of the SMP foam [13], in which case the low flow rate used in this study could be relevant. Regardless, testing under clinically relevant conditions is a necessary future step in the device development process, but is beyond the scope of this work.

The primary objective of this study was to illustrate the use of MRI as a tool in the preliminary stage of SMP foam device development using a simplified *in vitro *model. In this case, fluid velocity and temperature at several laser powers under a steady flow condition were acquired non-invasively. This type of experiment can be used to enhance the device design and deployment parameters without necessarily providing clinically relevant measurements. For example, experiments performed using devices with different foam densities would provide a means of elucidating the impact of foam density on the flow and temperature. Similarly, the thermal impact of different dye concentrations in the foam can also be quantified. By repeating the experiments at different flow rates, the effect of flow rate on the temperature can be gauged; this is important, for example, for evaluating methods of temporarily reducing the flow (e.g., baffle or balloon) to facilitate thermal actuation. The information provided can be used to refine the device and deployment parameters for subsequent testing under more clinically relevant conditions. In addition, since the experimental data is free from perturbation by invasive probes, it is particularly well-suited for development or validation of computational models of fluid and thermal dynamics.

Even with the limitations of steady-state measurements of flow velocity and temperature, the results were our first snapshot of the environment in the foam. The thermal result of reaching a maximum temperature change of 4°C at 7 W is not a surprise. We have previously described that, using simulations [15], flowing fluids (e.g. blood or water) are efficient at cooling the heated foams and distributing the thermal loads throughout the fluid volume. To date, however, we have had little intuition for the flow development in the foam. Fig. 3 shows the development of non-axial flows that are on the order of 50% of the axial flow rates. As we modify the structure of our open celled foams, these patterns will give us insight to local permeabilities or pockets of closed cells, for example. Also, as we move to anatomical geometries, the MRI flow data will permit characterization of flow in our treatment of aneurysms. Our ultimate goal is to use the MRI imaging to study the flow of blood, with and without anti-clotting agents, to help us in our design of the foam structure and deployment devices.

## Conclusions

Spatial maps of the steady-state fluid velocity and temperature change inside and outside a laser-heated SMP foam device in a simplified *in vitro *model were acquired using MRI techniques. Despite the relatively low temporal resolution (i.e., long image acquisition time) which prevented image acquisition during laser-induced expansion of the foam, the post-expansion images contain useful information from a device development standpoint, potentially enabling quantitative comparison of different device designs. The non-invasive nature of MRI allowed measurement of the temperature rise and flow without introducing probes that perturb the system; this feature is particularly useful for comparison to computational models. Though non-physiological conditions were used in this initial study, the utility of MRI in the development of a thermally-activated SMP foam device has been demonstrated.

## Competing interests

The authors declare that they have no competing interests.

## Authors' contributions

WS participated in the fabrication of the device, design and execution of the experiment, and writing of the manuscript. EG and JLH participated in the design and execution of the experiment and writing of the manuscript. TSW participated in the fabrication of the device. DJM participated in the design and supervision of the study. All authors read and approved the final manuscript.
